# Effect of Oil Species on the Viscoelastic Behavior of a Surfactant Film Formed at the Oil/Water Interface

**DOI:** 10.1021/acs.langmuir.5c00229

**Published:** 2025-05-21

**Authors:** Hiroki Kuwabara, Koji Tsuchiya, Kyosuke Arakawa, Yoshifumi Yamagata, Kenichi Sakai, Hideki Sakai

**Affiliations:** † Department of Pure and Applied Chemistry, Faculty of Science and Technology, 26413 Tokyo University of Science, 2641 Yamazaki, Noda, Chiba 278-8510, Japan; ‡ Department of R&D Center, Ikeda Mohando Corporation., Ltd., 16, Jinden, Kamiichimachi, Nakaniikawagun, Toyama 930-0365, Japan; § Research Institute for Science and Technology, 26413 Tokyo University of Science, 2641 Yamazaki, Noda, Chiba 278-8510, Japan; ∥ Anton Paar Japan K. K., Riverside Sumida 1Fl, 1-19-9 Tsutsumi-dori, Sumida-ku, Tokyo 131-0034, Japan

## Abstract

Clarifying the viscoelastic properties of oil/water interfacial films is important for evaluating the resistance of emulsions to coalescence. In recent years, strain-controlled rheometers with a bi-cone geometry have gained significant attention for measuring the viscoelasticity of liquid/liquid interfaces. In the present study, we sought to clarify the effect of oil species on the viscoelastic behavior of the oil/water interfacial film formed by a nonionic surfactant (Span 65) and correlate it with an emulsion's stability. A series of interfacial rheological measurements on saturated hydrocarbons with varying alkyl chain lengths as the oil phase showed that the elasticity of the oil/water interfacial film increased as the difference between the alkyl chain length of the oil phase and that of Span 65 increased. The stability of the water-in-oil emulsions prepared using each oil phase also improved with increasing alkyl chain length difference. These results demonstrated that viscoelastic parameters evaluated using this interfacial rheology are promising indicators for predicting the emulsion's stability. From the perspective of differences in the orientations of Span 65 and the oil phase at the interface, we also discussed the mechanism by which the viscoelastic behavior of the interfacial film differs depending on the alkyl chain length of the oil phase.

## Graphical abstract

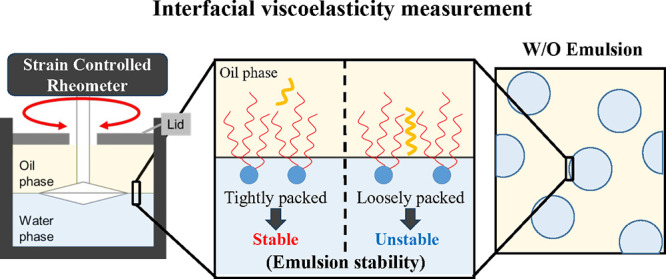

## Introduction

Emulsions are used in numerous industries, including pharmaceuticals, cosmetics, and food, owing to their ability to simultaneously incorporate water- and oil-soluble components in liquids. However, emulsions are thermodynamically metastable systems, with the emulsified droplets eventually coalescing and undergoing two-phase separation. Coalescence occurs when droplets fuse together as a result of the destruction of the interfacial film between the droplets. Therefore, a rigid emulsifier (surfactant) film formed at the oil/water interface will prevent further emulsion destabilization.^1^ Given this context, a quantitative evaluation of the strength of the oil/water interfacial film is expected to serve as an indicator for predicting the emulsions stability.

One method used to evaluate the physical properties of the surfactant film formed at the oil/water interface is to observe the microscopic fluidity of the interfacial film using a fluorescent reagent or a spin-label reagent with a radical-introduced amphiphilic molecule as a probe.^2–4^ However, this method has drawbacks, such as being unable to prove that the probe molecule is localized only at the interface being evaluated, so the physical properties of the actual surfactant molecules may not be accurately measured. Recently, we analyzed the structures of surfactant adsorption films formed at emulsion interfaces using small-angle X-ray scattering (SAXS) and indirect Fourier transformation analysis of its data and discussed the relationship between the structure of the interfacial film and the emulsions stability.^5^ Meanwhile, quantifying the macroscopic physical properties, such as the rheological properties, of oil/water interfacial films has been difficult.

An interfacial rheology system (IRS) comprising a strain-controlled rheometer with a bi-cone geometry has recently attracted attention for its ability to quantitatively evaluate the viscoelasticity of surfactant films formed at liquid/liquid (and gas/liquid) interfaces.^6^ Previous studies have investigated the emulsification properties and strength of oil/water interfacial films using polymers found in Arthrospira platensis protein extracts and biofilms formed by Gluconacetobacter xylinus.^7,8^ Meanwhile, only a few studies have evaluated the strength of oil/water interfacial films using low-molecular-weight surfactants that are widely used in industry.^9,10^ These studies have mainly focused on the nonionic surfactant sorbitan tristearate (Span 65), which is known to form rigid interfacial films, and either dodecane or silicon oil was used as the oil agent. To the extent of our knowledge, no other reports on the effects of other oil agents on the viscoelastic behavior of Span 65 films have been published. The emulsions stability is also influenced by the oil’s properties, so it is necessary to develop a systematic understanding of the effects of oil species on the viscoelastic behavior of surfactant films. To fill this research gap, in the present study, we used saturated hydrocarbon oils with varying chain lengths as oil agents to clarify the effect of oil species on the viscoelastic behavior of Span 65 films formed at the oil/water interface and examined its correlation with emulsion stability.

## Experimental Section

### Materials

Span 65 and sorbitan monostearate (Span 60) were purchased from Sigma-Aldrich. Scheme 1 depicts their molecular structures. The oils octane (≥98%), decane (≥99%), dodecane (≥99%), tetradecane (≥97%), and squalane (≥95%) were purchased from Fujifilm Wako Pure Chemical Co. (Osaka, Japan). All materials were used without further purification. It was confirmed by interfacial tension measurements that the impurities contained in squalane or other oils do not affect the interfacial chemical properties with water. Milli-Q water was used for all sample preparations.

**Scheme 1:**
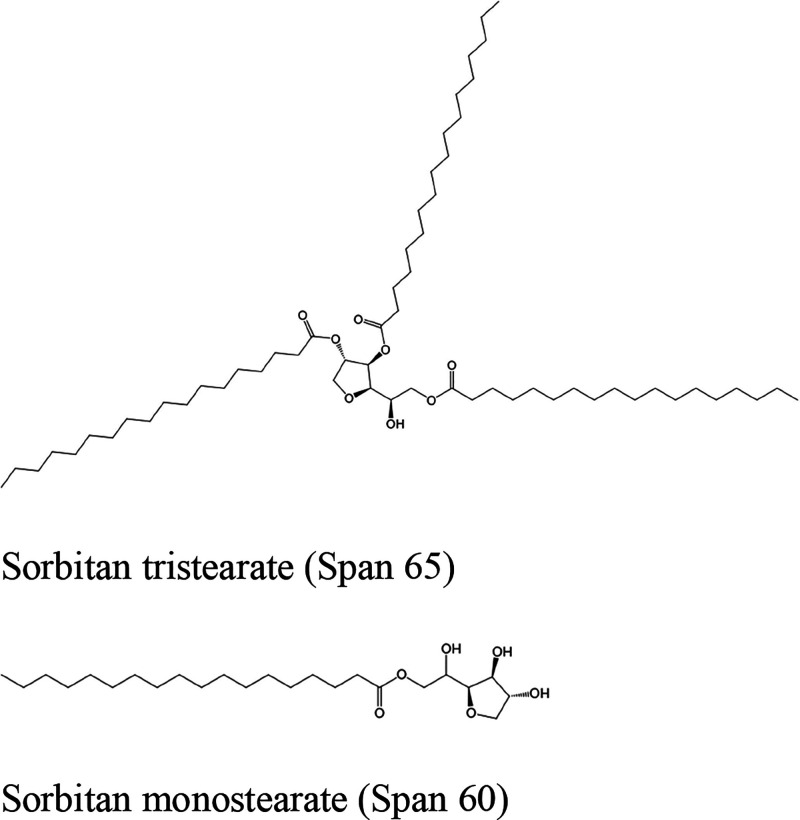
Emulsifiers Used in This Study

### Measurements of Interfacial Viscoelasticity at the Oil/Water Interface

Unlike conventional oscillatory rheology, which measures bulk viscoelasticity, interfacial rheology using a bi-cone specifically probes the two-dimensional viscoelastic properties of interfacial films. This technique isolates the viscoelastic response of the surfactant film from bulk effects, providing valuable insights into interfacial phenomena that cannot be obtained from conventional bulk rheology measurements. As shown in Figure 1, the interfacial rheology measurement was carried out using the MCR302 rheometer (Anton Paar GmbH, Austria) equipped with the IRS. The IRS consisted of a bi-cone (BiC 68-2×5, disk radius = 34.14 mm, cone angle = 5°, and cone penetration depth = 2.205 mm); a glass measuring cell (cup inner radius = 40.00 mm, cup height = 45.00 mm); and a heating/cooling jacket. The temperature was maintained at 25 °C using a Peltier plate.

**Figure 1:**
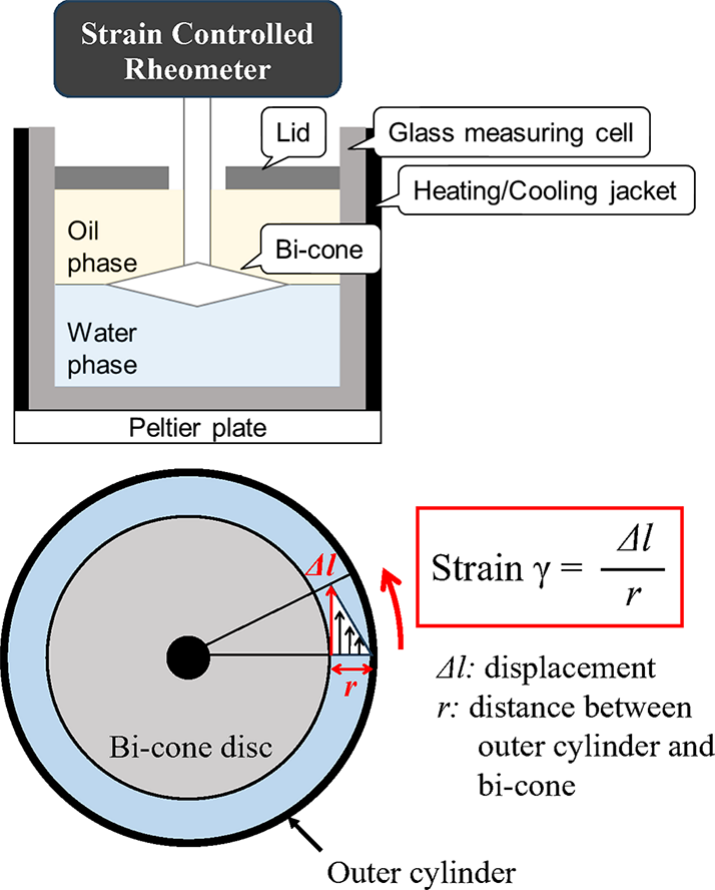
Schematic of interfacial rheology system (IRS).

The procedure for preparing the interfacial film at the oil/water interface was as follows: the aqueous phase was slowly poured into the glass measuring cell, the bi-cone was lowered until a change in normal force occurred, bringing the tip of the bi-cone into contact with the air/water interface. The bi-cone’s edge was lowered further to align it with the air/water interface, and then the oil phase was poured gently towards the bi-cone’s prop (or jig) using a pipette to avoid disturbing the interface.

The oil phases were prepared before conducting measurements by dissolving Span 65 in various oils to a concentration of 0.5 mmol dm^–3^ at 80 °C and then cooling to 25 °C. This concentration was selected for the interfacial viscoelasticity measurements to ensure consistency with previous studies.^9^

The film formed by the adsorption of Span 65 at the oil/water interface required time to stabilize; therefore, strain–sweep and frequency–sweep experiments were performed after confirming that the viscoelasticity parameters interfacial storage modulus *G*′ (Pam) and interfacial loss modulus *G*″ (Pam) had reached a steady state through time-sweep measurements, indicating stabilization of the Span 65 film.

### Method for Calculating Interfacial Viscoelastic Moduli

#### Derivation of the Viscoelastic Moduli Using Bi-Cone Geometry

The approach for determining the viscoelastic moduli *G*′ and *G*″ involves the following three steps:

(a) Application of Strain (γ):

A controlled sinusoidal strain (γ) was applied to the interfacial film using the bi-cone geometry. The strain is expressed as follows:

$$\gamma =\frac{\Delta l}{r}$$ 1

where *r* is the radial distance between the outer edge of the bi-cone and the inner wall of the glass measuring cell, Δ*l* is the horizontal displacement of the interface, as illustrated in Figure 1.

(b) Measurement of shear stress response and phase shift:

The shear stress (τ) was calculated based on the measured torque generated in response to the applied sinusoidal strain. Both the input strain and the output shear stress oscillated sinusoidally, with the phase shift (δ) determined as the time lag between the input strain signal and the output stress signal.

(c) Calculation of Viscoelastic Parameters:

The ratio of the maximum shear stress (τ_0_) to the maximum strain (γ_0_) defines the complex modulus (G*), which incorporates both elastic and viscous components. The viscoelastic moduli are then calculated by multiplying this ratio by the cosine or sine of the phase shift (δ), depending on whether the elastic or viscous component is considered, as shown in the following equations:

$$G^{\prime }=\frac{\tau_{0}}{\gamma_{0}}\cos\;\delta \;(\mathrm{Storage}\;\mathrm{modulus},\mathrm{representing}\;\mathrm{the}\;\mathrm{elastic}\;\mathrm{component})$$ 2

$$G^{\prime \prime }=\frac{\tau_{0}}{\gamma_{0}}\sin\;\delta \;(\mathrm{Loss}\;\mathrm{modulus},\mathrm{representing}\;\mathrm{the}\;\mathrm{viscous}\;\mathrm{component})$$ 3

#### Calculation of Interfacial Viscoelastic Moduli

The *G*′ (Pa) and *G*″ (Pa) obtained in the previous section contain viscoelastic information on the bulk phase in addition to that of the interface. Therefore, the influence of the bulk needs to be subtracted to obtain the interfacial viscoelastic moduli. The interfacial storage modulus (*G*′ (Pam)) and interfacial loss modulus (*G*″(Pam)) were calculated by solving the Navier-Stokes equations for the bulk phase velocity field with the Boussinesq-Scriven boundary condition, which describes interfacial stress and strain by considering the coupling between the interface and the bulk phase, even when their viscosities differ. This model is particularly relevant for interfacial rheology as it allows for the determination of interfacial viscoelastic moduli by incorporating interfacial viscosity and its influence on the bulk flow. The calculations were performed using a program that implements the iterative scheme developed by Sánchez-Puga et al.^11^

### Measurements of Interfacial Tension

This measurement was conducted to complement the interfacial rheology results and to obtain a better understanding of the molecular packing behavior of Span 65 at the oil/water interface. Interfacial tension was measured at 25°C using a Tracker tensiometer (Teclis Instruments, France) with the buoyant/rising drop method. An 18-gauge needle was used to form an oil droplet in the aqueous phase, and the interfacial tension was calculated using the Young–Laplace equation. The oil phase was prepared by dissolving Span 65 in various oils to a concentration of 0.5 mmol dm^–3^ to ensure consistency with the interfacial rheology measurements.

### Preparation of Water-in-Oil (W/O) Emulsions

W/O emulsions were prepared to investigate the correlation between emulsions stability and viscoelasticity parameters of the interfacial films. The oil phase was prepared by dissolving Span 65 in various alkanes to a concentration of 20 mmol dm^–3^ at 80 °C and then cooling to 25 °C (total volume of 4 mL). After adding water (6 mL volume) to the oil phase at 25 °C, the mixture was stirred for 10 min using a vortex mixer to prepare the W/O emulsions. Preliminary experiments indicated that emulsions with Span 65 concentrations of 20 mmol dm^–3^ or higher in the oil phase exhibited enough stability. Based on these results, 20 mmol dm^–3^ was selected as the standard concentration for the emulsion stability tests in this study.

## Results and Discussion

### Determination of Necessary Time for the Span 65 to Cover the Interface

We investigated the interfacial rheological properties of the interfacial film formed with surfactant (Span 65) by conducting time-, strain-, and frequency-sweep measurements. We first conducted time-sweep measurements to confirm that the viscoelasticity of the Span 65 film had reached a steady state, indicating that the interface had reached equilibrium. Strain-sweep measurements were then performed to determine the linear viscoelastic (LVE) region. Once the LVE was confirmed, frequency-sweep measurements were carried out at a fixed strain within this region to analyze the frequency dependence of the viscoelastic properties. All measurements were repeated two to three times, and enough reproducibility was confirmed. We also tried to measure the interfacial rheology of dodecane or squalane/water without adding Span 65, but we were unable to obtain the correct viscoelastic parameters because the torque was below the sensitivity of the device.

Figure 2 shows the results of time-sweep measurements of the Span 65 film formed at the oil/water interface. The measurements were conducted at 25 °C by applying a weak oscillation (γ: 0.05%, ω: 1 rad s^–1^) corresponding to the LVE. When using octane (red color plot) and squalane (yellow color plot) as the oil phase, the *G*′ (closed circle) was larger than the *G*″ (open circle), indicating that the film had elastic properties with *G*′ dominance. Both elastic moduli reached a steady state after ≈1500 s. Researchers have previously reported that the Span 65 film that forms at the air/water interface reaches a steady state within a few minutes,^12^ and a longer time is needed for a film to form at the oil/water interface. Meanwhile, when decane (blue color plot), dodecane (green color plot), and tetradecane (light blue color plot) were used as the oil phase, the film showed *G*″-dominant viscous properties immediately after the measurement, and then changed to a *G*′-dominant elastic film over time.

**Figure 2:**
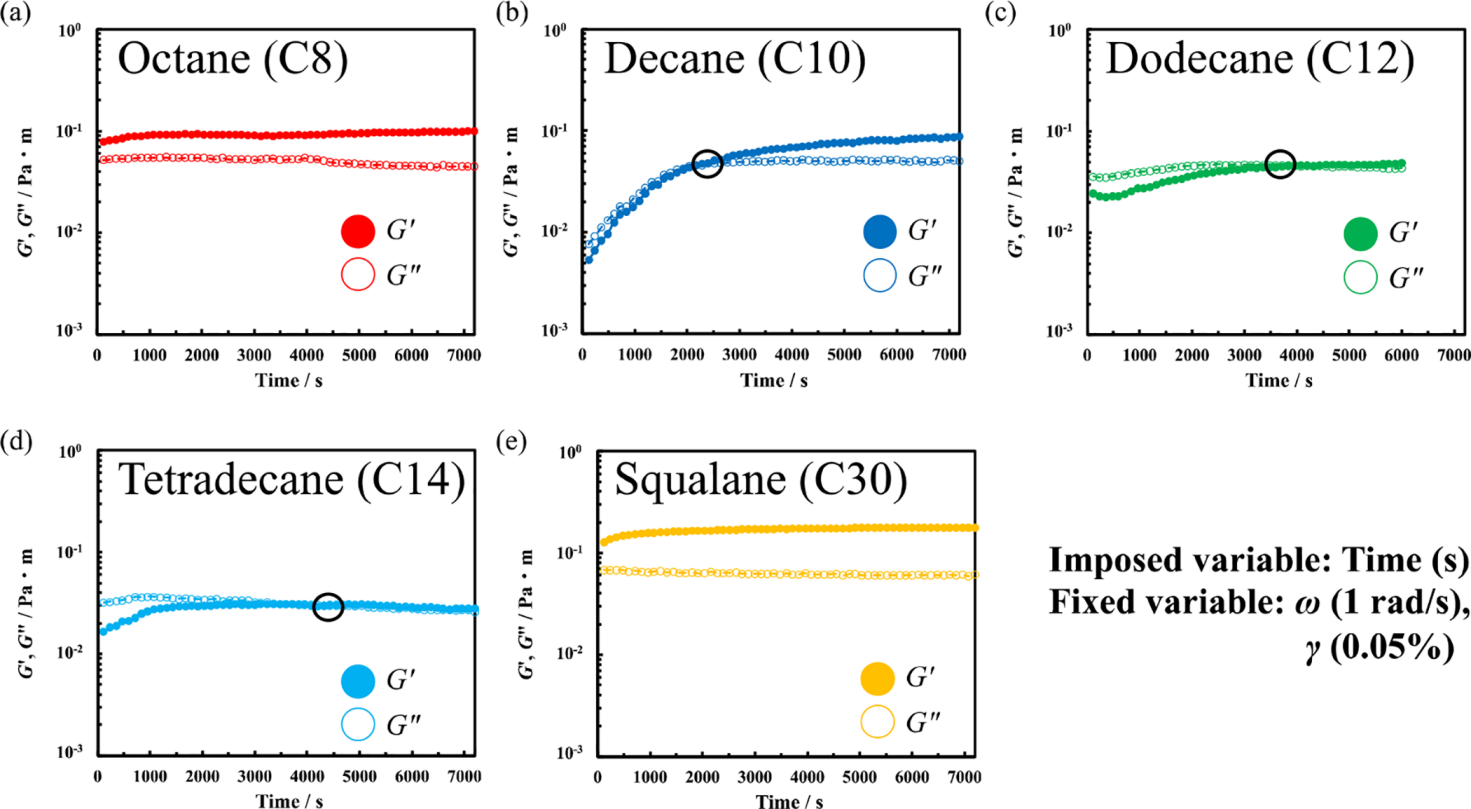
Interfacial storage modulus (*G*′; closed circles) and loss modulus (*G*″; open circles) of Span 65 films formed at the oil/water interface using different oil species (a) octane, (b) decane, (c) dodecane, (d) tetradecane and (e) squalane as a function of time at 25 °C (γ; 0.05%, ω; 1 rad s^–1^).

The time for *G*′ and *G*″ crossover, circled in black in Figure 2, increased as the hydrocarbon chain length increased from decane (C_10_H_22_) to tetradecane (C_14_H_30_). Therefore, longer hydrocarbon chain lengths (from octane to tetradecane) required more time to form the elastic film. However, contrary to this trend, the time required for elastic film formation was shorter for squalane, which had an even longer hydrocarbon chain length (C_30_H_62_ with branched chain).

Additionally, in the steady state for Span 65 films using octane, decane, and squalane, *G*′ was enough larger than *G*″, and elastic films were formed. Meanwhile, dodecane and tetradecane had similar *G*′ and *G*″ values, forming films with both elastic and viscous properties, similar to that observed in entangled polymer solutions near the sol–gel transition.^13,14^ Therefore, oil agents that required shorter time periods to form an elastic Span 65 film tended to form films with higher elasticity.

### Determination of the Linear Viscoelastic (LVE) Region

We confirmed that the interfacial rheology measurements were conducted in the LVE region by conducting strain-sweep measurements (ω = 1 rad s^–1^) at 25 °C. Figure 3 shows the strain-sweep measurement results for a Span 65 film formed at the oil/water interface. Error bars in panel (c) of Figure 3 is shown in Figure S1 in the Supporting Information.

**Figure 3:**
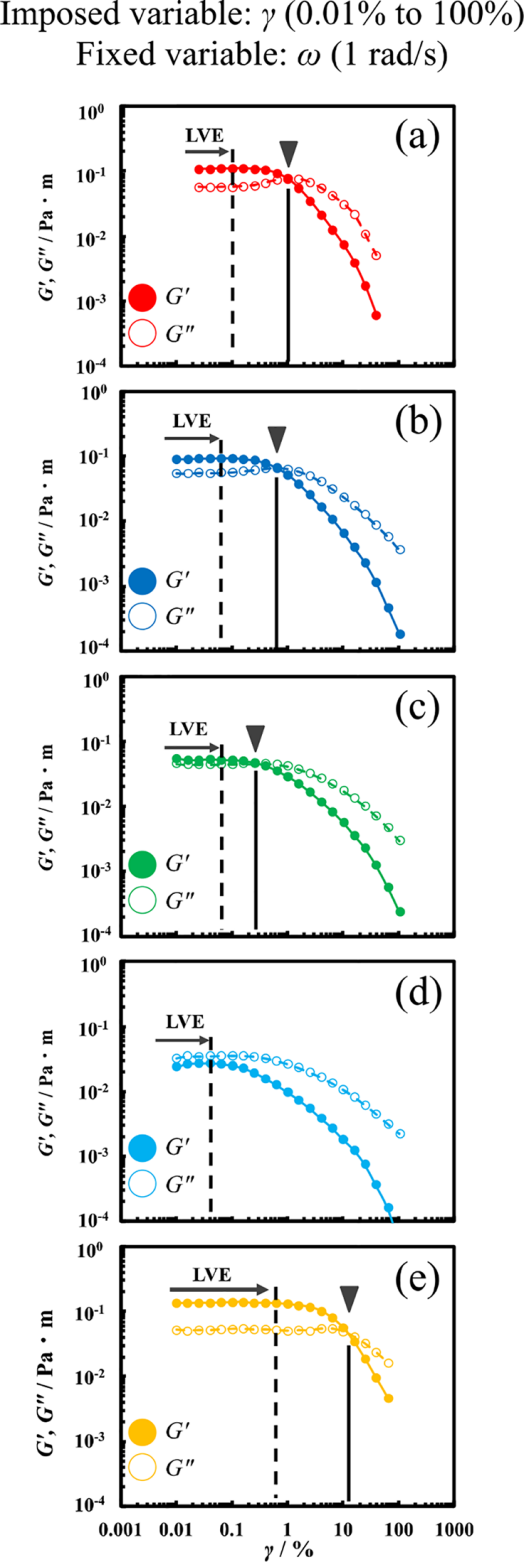
Interfacial storage modulus (*G*′; closed circles) and loss modulus (*G*″; open circles) of Span 65 films formed at the oil/water interface using different oil species (a) octane, (b) decane, (c) dodecane, (d) tetradecane and (e) squalane as a function of the strain amplitude at 25 °C (ω; 1 rad s^–1^).

Oil agents other than tetradecane exhibited an interfacial rheological behavior such that *G*′ was dominant in the low-strain region, and increasing strain values resulted in the disordering of the Span 65 film and *G*″ becoming dominant. This behavior is similar to that reported in normal (three-dimensional) rheology, such as entangled polymer solutions and worm-like micelles.^15,16^

Additionally, an increasing alkyl chain length from octane to tetradecane resulted in a shift in the LVE region to the low-strain region. Meanwhile, the LVE region of squalane, which has an even longer hydrocarbon chain length, shifted to the high-strain side. Similarly, the longer linear alkyl chains of octane to dodecane resulted in the crossover point of *G*′ and *G*″ shifting to the low-strain region, while the overlapping point of *G*′ and *G*″ for squalane shifted to the high-strain region. Therefore, alkyl chain lengths from octane to tetradecane resulted in a weaker structure of the Span 65 film formed at the oil/water interface with respect to strain displacement, while the film structure was stronger for squalane. These results correlated with the time-sweep measurement results. Previous research has reported that two-dimensional Span 65 films behave as viscoelastic bodies in systems using dodecane.^9,10^ However, no previous study has reported that changing the hydrocarbon length of the oil agent results in significant changes in the interfacial rheological behavior of Span 65 films formed at the oil/water interface.

Figure 4 shows the loss tangent (tan δ) in the LVE region of the strain-sweep measurement plotted with respect to the hydrocarbon chain length of the oil phase. Here, tan δ can be expressed as the ratio of *G*″/*G*′, so a smaller tan δ indicates that the film is an elastic film with *G*′ dominance. The tan δ value of the Span 65 film in a static state without structural destruction becomes higher with longer chain length, ranging from octane to tetradecane, and a linear relationship on a logarithmic scale was found for alkyl chain lengths in the range of 8 to 14. Meanwhile, the tan δ value of squalane, which has 30 carbon atoms but a six-branch structure, deviated considerably from a straight line and exhibited high elasticity. In this measurement, tan δ of interfacial films tended to increase as the hydrocarbon chain length increased. Meanwhile, the viscosity of the oil phase (bulk phase) dissolving Span 65 were Newtonian fluids and the viscosity increased with increasing hydrocarbon chain length of the oils (Figure S2). Therefore, the results of the interfacial rheology measurements in this study were not affected by the bulk viscosity and the analysis was carried out appropriately.

**Figure 4:**
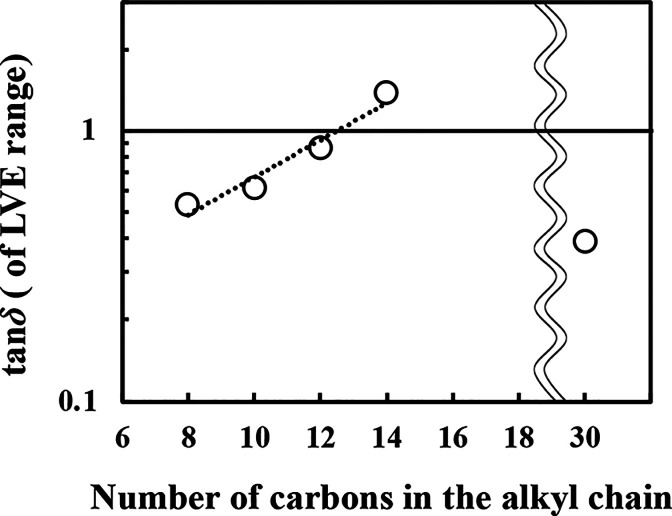
Tan δ of strain-sweep measurements of Span 65 films formed at the oil/water interface using different oil species at 25 °C, represented as a function of hydrocarbon chain length.

The viscoelastic behavior of the Span 65 film is not due to chemical crosslinking, but rather to temporary physical crosslinking as a result of hydrogen bonds between hydrophilic groups and van der Waals interactions between hydrophobic groups.^9,12^ Figure 5 shows the strain-sweep measurement results of Span 65 and Span 60 at the dodecane/water interface. The interfacial film formed by Span 60 had a significantly lower viscoelasticity than that by Span 65. This difference is presumed to be due to the fact that Span 60 has fewer hydrophobic groups (one stearyl group) than Span 65 (three stearyl groups). In other words, the contribution of the van der Waals attractive interactions of the hydrophobic group (stearyl group) is stronger than that of hydrogen bonds between hydrophilic groups (sorbitan) in the formation of the *G*′-dominated elastic film, which is similar to the results of the films of Span 65 and Span 60 formed at the air/water interface.^12^ In addition to these interactions, other mechanisms, such as steric hindrance arising from tighter molecular packing, may also contribute to the observed viscoelastic behavior.

**Figure 5:**
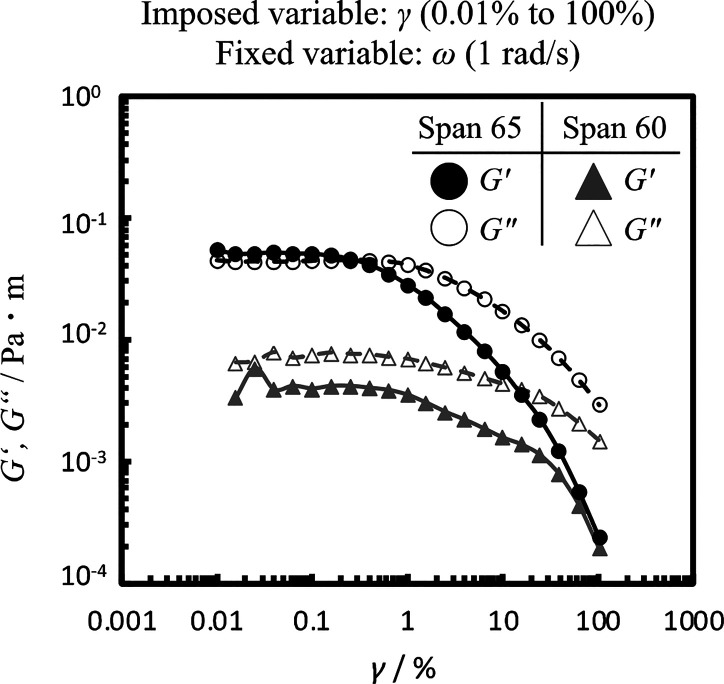
Interfacial storage modulus of Span 65 (circles) and Span 60 (triangles) films formed at the dodecane/water interface as a function of the strain amplitude at 25 °C (ω; 1 rad s^–1^).

### Determination of Relaxation Time

Figure 6 shows the frequency-sweep measurements of a Span 65 film formed at the oil/water interface (γ = 0.05%) in the LVE region. The *G*′ of squalane was consistently large within the measurement frequency range, forming an elasticity-dominated film. Meanwhile, hydrocarbon oils with alkyl chain lengths ranging from octane to tetradecane exhibited a crossover between *G*′ and *G*″ at a specific frequency (ω_co_). In other words, these oils behaved as an elastic film with *G*′ dominance for the higher frequency region above ω_co_, but as a viscous film with *G*″ dominance for the lower one. The results also indicate that they do not follow the Maxwell model with a single relaxation time, but rather have multiple relaxation mechanisms. This relaxation behavior may be attributed to the interfacial surfactant molecules undergoing displacement in response to strain and gradually returning to their original orientations as the deformation is released. The presence of multiple relaxation mechanisms suggests that the interfacial film may not be structurally uniform.

**Figure 6:**
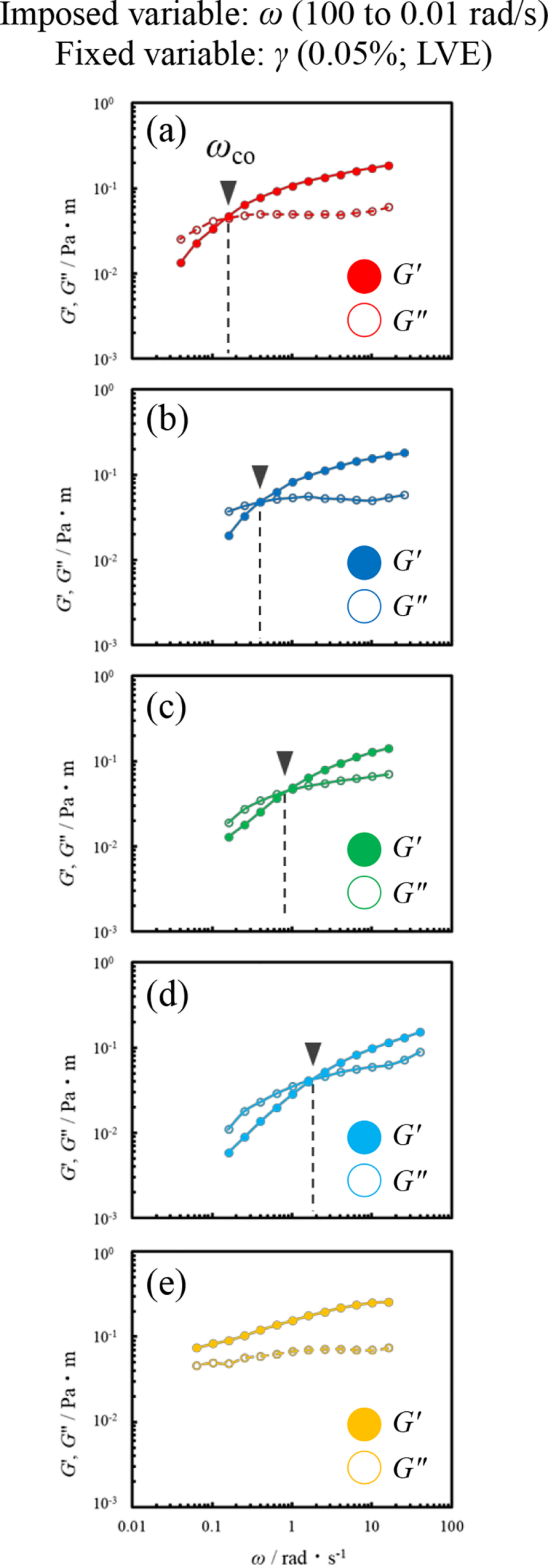
Interfacial storage modulus (*G*′; closed circles) and loss modulus (*G*″; open circles) of Span 65 films formed at the oil/water interface using different oil species (a) octane, (b) decane, (c) dodecane, (d) tetradecane and (e) squalane as a function of the frequency at 25 °C (γ; 0.05%).

Furthermore, an increasing hydrocarbon chain length from octane to tetradecane resulted in ω_co_ shifting to the higher frequency region. Figure 7 shows the average relaxation time τ = 1/ω_co_ as a function of hydrocarbon chain length. Increasing the hydrocarbon chain length from octane to tetradecane correspondingly decreased the average relaxation time, indicating that the Span 65 film forms temporary physical crosslinking which only persist over shorter times when an oil with a longer hydrocarbon chain is used.

**Figure 7:**
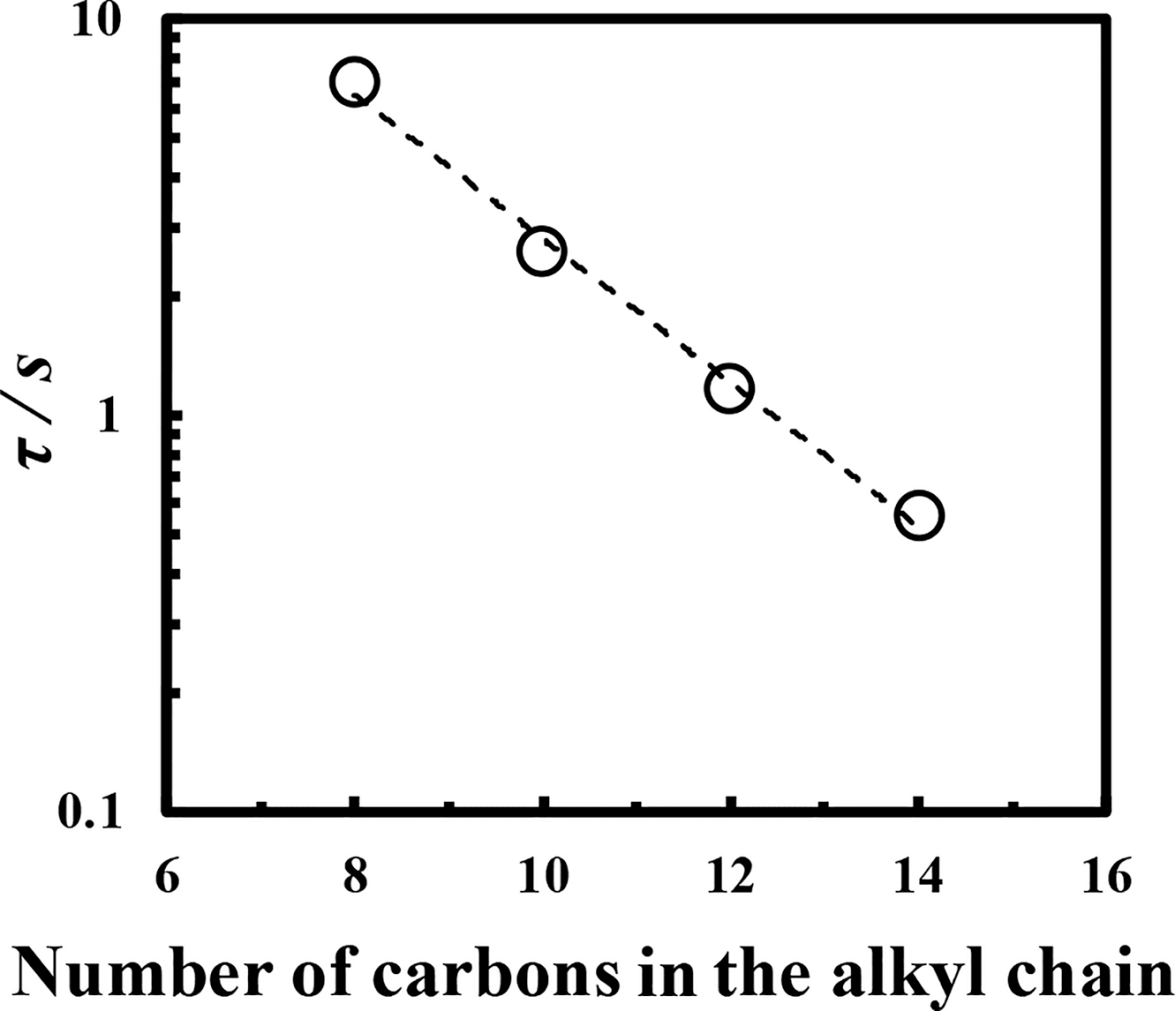
Relaxation times (τ) of Span 65 films formed at the oil/water interface using different oil species at 25 °C, represented as a function of hydrocarbon chain length.

The above-mentioned results of the interfacial rheology experiments, including the time-, strain-, and frequency-sweep measurements, indicate that a larger difference between the alkyl chain length of Span 65 (stearyl group, *n* = 18) and alkyl chain of the oil agent leads to the formation of a more elastic interfacial film. As suggested by the strain sweep results for Span 65 and Span 60 (Figure 5), the van der Waals attractive interactions between the tristearyl chains of Span 65 may play an important role in the formation of a highly elastic film. It was inferred that tetradecane, which has an alkyl chain length close to that of the stearyl group of Span 65, had the strongest van der Waals interaction with the stearyl group of Span 65 among the investigated oil agents. Therefore, as shown in Figure 8a, tetradecane penetrates between Span 65 molecules to form a less dense film, indicating that an elastic Span 65 film was not formed. In addition, in the case of hexadecane, which is predicted to interact more strongly with the alkyl chains of Span 65 than tetradecane, the value of interfacial viscoelasticity was so low that it could not be reproduced. Meanwhile, as shown in Figure 8b, the oil agent with a short alkyl chain length does not weaken the van der Waals interaction among the stearyl groups in Span 65, indicating that the oil agent was less easily incorporated into the interfacial film. As a result, it was thought that the interaction between Span 65 molecules became stronger, forming an elastic film.

**Figure 8:**
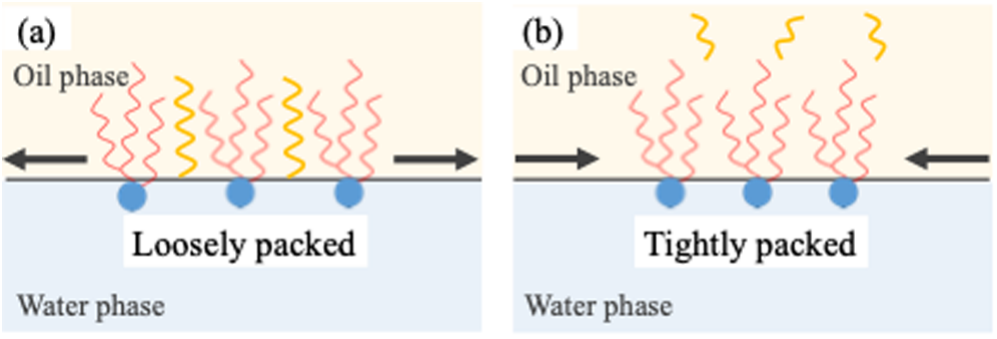
Schematic of Span 65 film formed at the oil/water interface when the difference between the alkyl chain lengths of Span 65 (C18) and hydrocarbons (oil phase) is (a) large and (b) small.

To demonstrate the validity of this interpretation, interfacial tension measurements were performed. As shown in Figure 9, the interfacial tension showed a decrease with decreasing alkyl chain length of the oil phase, indicating a higher adsorption density of Span 65 at the interface. Additionally, the interfacial tension of squalane was lower than that of octane. Squalane has the alkyl chain much longer than that of the stearyl group of Span 65 and also a methyl-branched chain, making it difficult to penetrate between the Span 65 molecules.

**Figure 9:**
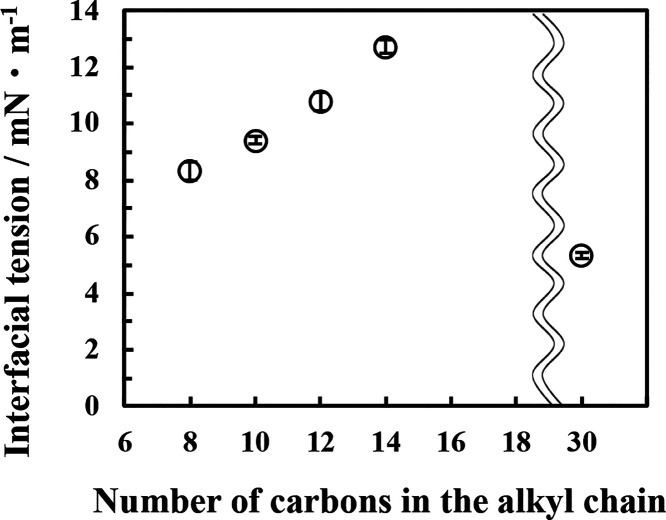
Interfacial tension between water and the oil phase with various alkyl chain length dissolving Span 65 (0.5 mmol dm^–3^) at 25 °C. Data are presented as mean ± standard deviation from three independent measurements.

Such differences in packing of the emulsifier due to variations in alkyl chain length between the oil phase and the emulsifier have been reported in a previous study.^17^ Hayashi et al. conducted interface pressure-area isotherm measurements of the dipalmitoyl glycerophosphoethanolamine (DPPE) at the alkane/water interface. They showed that an oil agent chain length that was closer to the alkyl chain length (hexadecyl group) of DPPE resulted in deeper oil agent penetration between the DPPE molecules and a greater molecular area occupied by DPPE. This finding supports the above discussion.

### Relationship between Viscoelastic Behavior of Interfacial Film and Emulsion Stability

Figure 10 shows images of W/O emulsions prepared using various oil agents over the time, as well as their emulsion stability and the correlation with oil/water interfacial viscoelastic parameter (tan δ). The stability of the emulsions was evaluated based on the proportion of the remaining emulsified phase 6 h after preparation (shown in Figure S3). We observed that as the hydrocarbon chain length increased from octane to tetradecane, the separation amount of the oil and water phases increased, and the emulsion was destabilized. On the other hand, the stability of emulsion prepared with squalane bearing much longer hydrocarbon chain was higher. In general, the separation of the oil and water phases is caused by the coalescence of dispersed droplets followed by creaming. In fact, optical microscopy observation of the prepared emulsion after 6 h revealed that in tetradecane, which exhibited a lower interfacial elasticity, coalescence of droplets progressed, resulting in the formation of larger droplets. In contrast, in octane and squalane, which exhibited higher interfacial elasticity, coalescence was suppressed, and individual droplets remained smaller (shown in Figure S4). Therefore, it was suggested that the higher the viscoelastic parameters of the oil/water interfacial film, the higher the resistance to coalescence of the emulsion.

**Figure 10:**
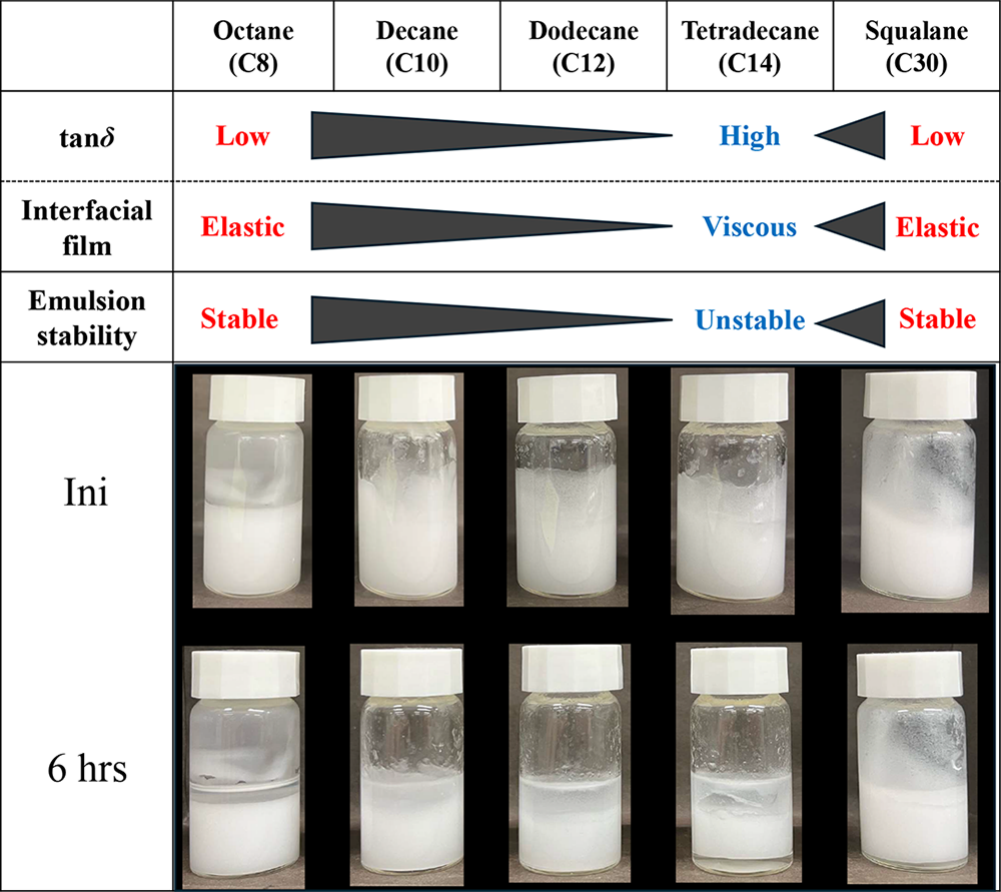
Correlation between viscoelasticity parameters of the oil/water interfacial films and the emulsions stability.

These results suggest that the viscoelastic properties of interfacial films are closely linked to emulsion stability. This study demonstrates that interfacial rheology measurements enable quantitative evaluation of film elasticity, which directly reflects the interfacial film’s resistance to droplet coalescence. Although other factors, such as continuous phase viscosity and electrostatic interactions, should also be considered, the present approach offers a useful indicator for predicting emulsion stability. We believe this knowledge will greatly contribute to the efficiency of emulsion formulation development in various industrial fields, such as cosmetics, food, and pharmaceuticals.

## Conclusions

We found that a larger difference between the alkyl chain length of the saturated hydrocarbon used as the oil agent and that of the Span 65 used as the emulsifier resulted in higher elasticity of the Span 65 film formed at the oil/water interface, as well as lower interfacial tension and increased emulsions stability of the W/O emulsion. These results indicate that the viscoelasticity parameters evaluated using interfacial rheology measurements are promising indicators for predicting the long-term coalescence stability of emulsions. We also showed that the physicochemical properties of the interfacial film can be controlled via the choice of oil agent. We anticipate that the results of this study will serve as a guideline for selecting an appropriate oil agent when designing emulsion formulations.

## Supplementary Material



## Notes

Error bars for strain-sweep measurements shown in panel (c) of Figure 3; measurements of the bulk viscosity of the oil phase dissolving Span 65; proportion of emulsified phase remaining after 6 h; and optical microscopic observations of the emulsion after 6 h (PDF)
