# Supplementary material for: Effect of Oil Species on the Viscoelastic Behavior of a Surfactant Film Formed at the Oil/Water Interface
Source: Langmuir. 2025 May 21;41(21):12914–21. doi: 10.1021/acs.langmuir.5c00229 (PMC12139030; doi:10.1021/acs.langmuir.5c00229)
Supplement: Supplementary file 1 [file la5c00229_si_001.pdf]

Effect of Oil Species on the Viscoelastic Behavior of Surfactant Film Formed at Oil/Water Interface

Hiroki Kuwabara[a,b,*], Koji Tsuchiya[c], Kyosuke Arakawa[a], Yoshifumi Yamagata[cd],

Kenichi Sakai[ac], and Hideki Sakai[a,c,*]

[a]Department of Pure and Applied Chemistry, Faculty of Science and Technology, Tokyo University of Science, 2641 Yamazaki, Noda, Chiba 278-8510, Japan

[b]Department of R&D Center, Ikeda Mohando Corporation., Ltd., 16, Jinden, Kamiichimachi, Nakaniikawagun, Toyama 930-0365 Japan

[c]Research Institute for Science and Technology, Tokyo University of Science, 2641 Yamazaki, Noda, Chiba 278-8510, Japan

[d]Anton Paar Japan K. K., Riverside Sumida 1Fl, Tsutsumi-dori, Sumida-ku, Tokyo, 1-19-9, Japan

* Corresponding author E-mail:

* Hideki Sakai: hisakai@rs.tus.ac.jp

* Hiroki Kuwabara: kuwabara.h@ikedamohando.co.jp

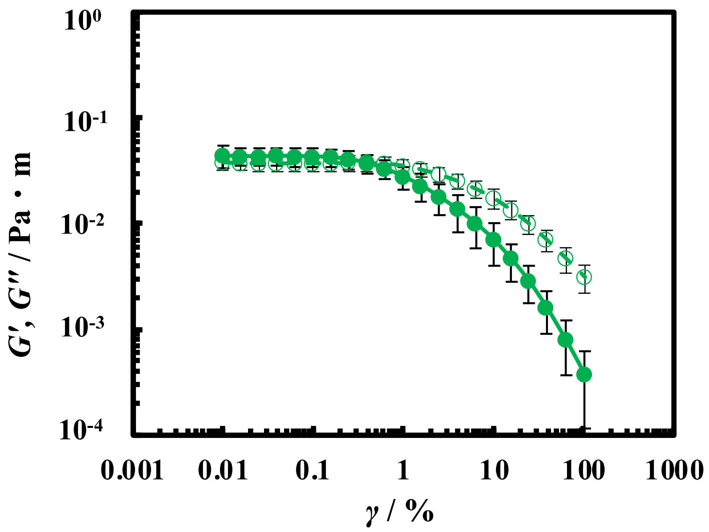

Figure S1. Interfacial storage modulus (G') and loss modulus (G'') of the Span 65 film at the dodecane/water interface as a function of strain amplitude at 25 °C (ω = 1 rad·s⁻¹). Error bars represent the standard deviation from three independent measurements.

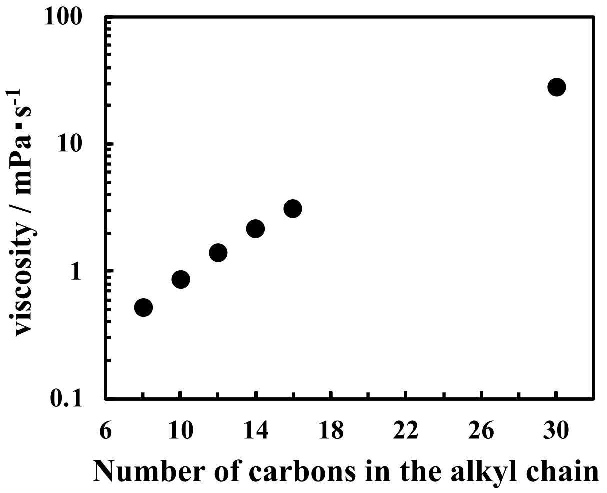

Figure S2. Measurements of the bulk viscosity of the oil phase dissolving Span 65 (0.5 mmol dm^3) were conducted at 25 °C.

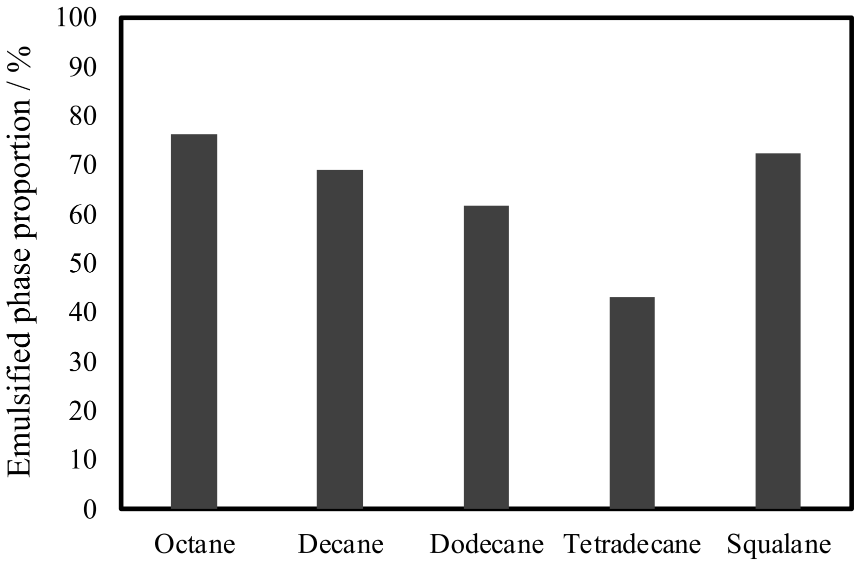

Figure S3. Emulsified phase proportion remaining 6 hours after emulsification with various oils.

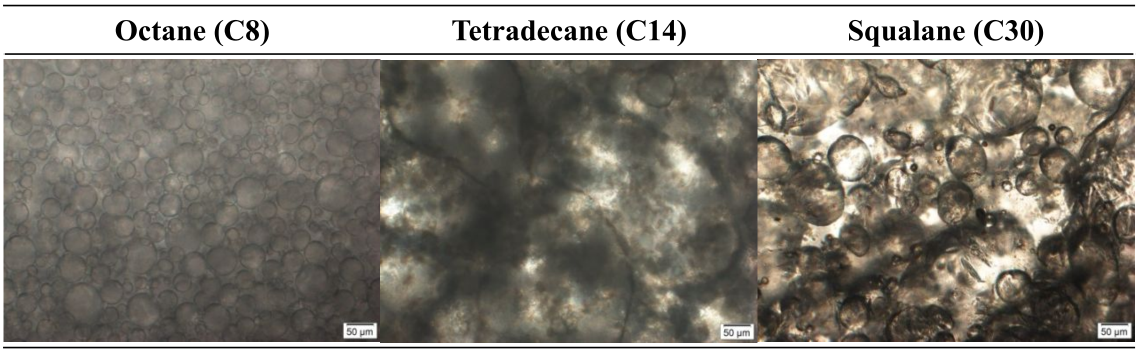

Figure S4. Optical microscopic observation of the emulsions after 6 hours with various oils.
